# Quantifying the Timescale and Strength of Southern Hemisphere Intraseasonal Stratosphere‐troposphere Coupling

**DOI:** 10.1029/2019GL084763

**Published:** 2019-11-20

**Authors:** Elena Saggioro, Theodore G. Shepherd

**Affiliations:** ^1^ Department of Mathematics and Statistics University of Reading Reading UK; ^2^ Department of Meteorology University of Reading Reading UK

**Keywords:** Time‐series Causal Network, Stratosphere‐Troposphere coupling, intra‐seasonal transition, Southern Hemisphere zonal circulation, zonal circulation trends, autocorrelation timescale

## Abstract

The Southern Hemisphere zonal circulation manifests a downward influence of the stratosphere on the troposphere from late spring to early summer. However, the strength and timescale of the connection, given the stratospheric state, have not been explicitly quantified. Here, SH zonal wind reanalysis time series are analyzed with a methodology designed to detect the minimal set of statistical predictors of multiple interacting variables via conditional independence tests. Our results confirm from data that the variability of the stratospheric polar vortex is a predictor of the tropospheric eddy‐driven jet between September and January. The vortex variability explains about 40% of monthly mean jet variability at a lead time of 1 month and can entirely account for the observed jet persistence. Our statistical model can quantitatively connect the multidecadal trends observed in the vortex and jet during the satellite era. This shows how short‐term variability can help understand statistical links in long‐term changes.

## Key Points

- Autocorrelation of stratospheric polar vortex variability inflates persistence of cross correlations with the tropospheric eddy‐driven jet
- A conditional analysis identifies the vortex as statistical predictor for 40% of monthly jet variability in spring/summer
- The inferred statistical model explains the enhanced jet persistence and connects the vortex and jet trends observed in the satellite era

## 1 Introduction

The Southern Hemisphere (SH) stratospheric and tropospheric extratropical circulation variabilities are strongly coupled during the spring‐to‐summer transition (S‐T coupling). The coupling is manifested in the vertical structure of large‐scale patterns of climate variability, such as the Southern Annular Mode (SAM) (Thompson & Wallace, 2000), or of the anomalous zonal flow (Kuroda & Kodera, 1998). Its intensity maximizes between late spring and early summer, generally lasting until the late spring stratospheric polar vortex breakdown (BD). On intraseasonal timescales, the coupling's observational fingerprint is a downward propagating structure of the anomalies (Kuroda & Kodera, 2001; Thompson et al., 2005). A similar phenomenon is seen in the Northern Hemisphere (Baldwin & Dunkerton, 2001), although it is less tied to the seasonal cycle.

Modeling studies of the SH have confirmed the stratospheric origin of the downward propagation. Arguably, the strongest evidence is provided by studies of the late 20th century ozone depletion. Multidecadal trends of the SH tropospheric circulation, in particular of the eddy‐driven jet, have been unequivocally traced back to the stratospheric ozone decline, reflected in a delay in the vortex BD (McLandress et al., 2010, 2011). Such a tropospheric response to an exogenous stratospheric perturbation can be regarded as causal in the physical sense of the term. Other modeling studies show a clear sign of increased tropospheric information conditional on the stratospheric state on a variety of timescales (Byrne et al., 2019; Ceppi & Shepherd, 2019; Seviour et al., 2014).

Considerable interest has focused on the enhanced autocorrelation timescale of the tropospheric SAM during late spring/early summer. Several authors (Gerber et al., 2010; Thompson et al., 2005) have suggested that this reflects the stratospheric influence at this time of year, but a theoretical model providing a quantification of such a connection has been lacking. Using a climate model, Simpson et al. (2011) showed that suppressing stratospheric variability in spring cuts the autocorrelation timescale of the tropospheric SAM by one‐half to about 2 weeks, but no further exploration of this interesting result followed. Instead, most studies have interpreted the enhanced SAM autocorrelation timescale in terms of purely tropospheric eddy‐feedback processes (Lorenz & Hartmann, 2001; Simpson et al., 2013).

Observational studies have shown that the vortex BD plays an organizing role for tropospheric circulation anomalies on weekly to monthly timescales (Black & McDaniel, 2007; Byrne et al., 2017; Hio & Yoden, 2005). Estimates of such potential for predictability, in terms of strength and timescale of the signal, have been quantified with lagged correlations (Byrne & Shepherd, 2018; Gerber et al., 2010; Graversen & Christiansen, 2003) or regression patterns (Thompson et al., 2005). These two methods are widely employed to identify statistical predictors (Wang et al., 2017). However, their results can be biased by the effect of autocorrelations (McGraw & Barnes, 2018) and are not designed to distinguish direct from indirect effects (Ebert‐Uphoff & Deng, 2012). More complex machine learning techniques have recently been adopted (Minokhin et al., 2017) to overcome those shortcomings, but such methods often lack physical interpretation (Runge et al., 2019).

In this work, we propose a theoretically based quantification of the predictor‐predictand relationship between observed vortex and jet anomalies for the spring‐to‐summer transition. Specifically, following Granger (1969), we ask how much additional information the stratosphere is providing to the knowledge of the state of the troposphere. A natural framework for such an analysis is that of time series causal networks (Ebert‐Uphoff & Deng, 2012; Runge et al., 2014). Causal networks are made of links connecting conditionally dependent variables, therefore distinguishing direct from indirect effects by design (Pearl, 2009). Their time series version allows to identify the specific timescale of a link and to handle autocorrelations (Kretschmer et al., 2016). As a result, we can identify the relevant timescale and quantify the strength of the downward connection despite the confounding effect of the vortex and jet autocorrelations. In order to avoid misleading interpretations that can arise when applying this mathematical method to a system governed by physical laws, we use the term predictor‐predictand pair rather than causal link.

## 2 Data

The primary data used is the four‐times‐daily wind from the ERA‐Interim reanalysis, available on an N128 Gaussian grid and 37 pressure levels (1000–1 hPa) (Dee et al., 2011). The period considered is 1 June 1979 to 30 April 2018 (39 spring‐to‐summer transitions). We define the vortex strength (PoV) using the daily‐mean zonal‐mean zonal wind ([*u*]) at 50 hPa and 60°S (Black & McDaniel, 2007; Byrne et al., 2017; Ceppi & Shepherd, 2019). This choice is considered to be robust due to the strong barotropic structure and temporal coherence of the vortex intraseasonal variability. The eddy‐driven jet strength at high latitudes (Jet) is defined as the sine‐weighted integral of [*u*] at 850 hPa between 50 and 65°S. By geostrophic balance, Jet is proportional to the semi‐annual oscillation (SAO) and eddy‐driven jet (EDJ) measures in Bracegirdle (2011) and Byrne and Shepherd (2018) respectively, both of which are defined as the difference in zonal‐mean sea‐level pressure between 50 and 65°S. Anomalies are computed by removing the climatology and are used in all analysis. The *n*‐days average of a daily time series is performed on nonoverlapping blocks of length *n*. The season 2002–2003 is discarded due to its outlier nature of being the only SH stratospheric sudden warming on record. The date of the vortex BD is computed following Black and McDaniel (2007). Only the first parts of our two time series are significantly trending; thus, detrending is uncertain (see Figures 3e and 3f). Since the vortex trend is mostly due to a delayed BD, conditioning the time window of the analysis around that date acts effectively as a detrending. With this BD dependent analysis, we recover almost identical results on the timescales of interest (compare Figures 2c and 2d); thus, avoiding explicit detrending does not impact the results.

## 3 Methods

**Figure 1:**
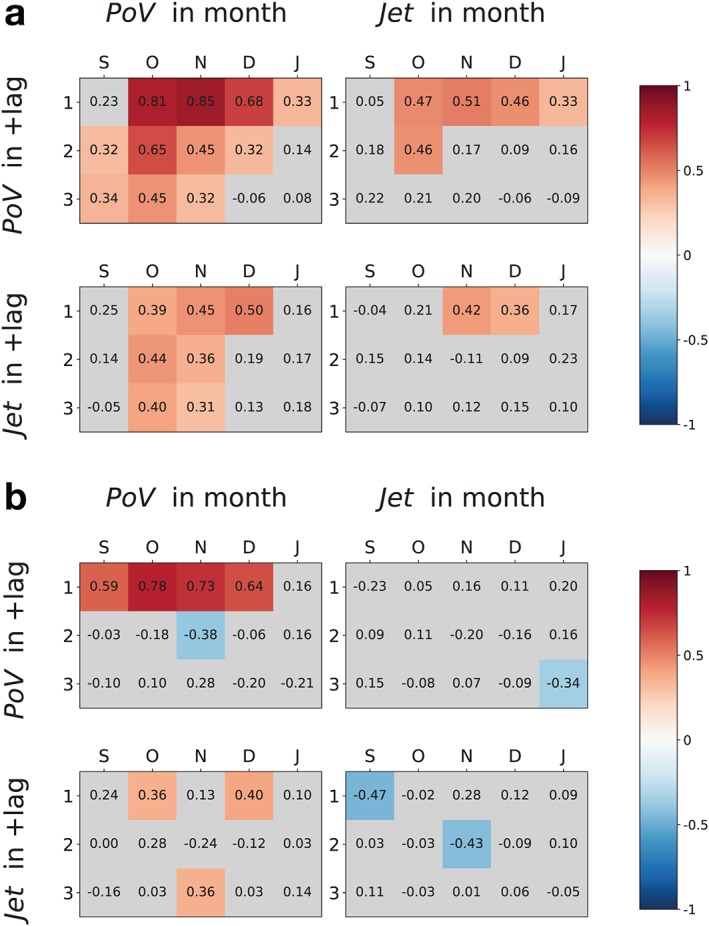
(a) correlation and (b) partial correlation *ρ* ^*MIT*^ matrices for PoV and Jet. In each subfigure, left panels show coefficients between monthly values of PoV and either (top) PoV or (bottom) Jet lagged by 1 to 3 months. Right panels show the same but for monthly values of Jet. If the *p* value is larger than 5% in a one‐tailed Student *t* distribution of 38 samples, the color of the entry is grey; otherwise, the color is a visual analog of the correlation coefficient.

A desirable property for a statistical predictor *X*_*t*_ is to provide information on its predictand *Y*_*t*+*τ*_ that is not found in any other variable of the system at a previous time, this set being indicated with *S*_*t′*<*t*+*τ*_. For linear systems, this property translates into the partial correlation coefficient *ρ*(*X*_*t*_,*Y*_*t*+*τ*_|*S*_*t′*<*t*+*τ*_) being significantly different from zero (Spirtes & Glymour, 1991). Partial correlation is the correlation between the residuals of two separate linear regressions of *X* and *Y* onto *S*. The causal network analysis starts with the null hypothesis that any pair of lagged variables (*X*_*t*_,*Y*_*t*+*τ*_) is potentially a predictor‐predictand pair, including (*X*_*t*_,*X*_*t*+*τ*_). For each pair, the partial correlation is computed together with its *p* value (according to a two‐tailed Student *t* distribution, except for Figure 1). The associated connection is rejected if the *p* value is larger than a desired significance level, *α*. The remaining connections constitute the time series causal network, that is, the set of lag‐specific statistical predictor‐predictand pairs.

Usually, the set *S* is prohibitively high dimensional to implement such a computation. Network theory allows to replace it with a suitable subset *Z* ^*MIT*^ without loss of generality (Runge et al., 2012) (see Text S1 of the supporting information). MIT stands for Momentary Information Transfer to highlight that the corresponding partial correlation, *ρ* ^*MIT*^, quantifies the unmediated dependence between two lagged variables. In particular, *Z* ^*MIT*^ may include the lagged‐in‐the‐past version of both variables and thus can remove biases induced by autocorrelations, together with other indirect effects (Runge, 2018). We stress that a link in the network represents a predictor‐predictand pair within the context of the data set: including additional variables could lead to a different set of optimal predictors (Ebert‐Uphoff & Deng, 2012). Thus, the initial choice of variables relies on expert (i.e., physical) knowledge. As the terms predictor/predictand are usually intended in a contextual sense, we adopt this terminology instead of the (a‐contextual) terms cause/effect.

The maximum lag tested for connection, *τ*_*max*_, and the significance level for link rejection, *α*, are specified in each part of the analysis. All causal analysis was performed with the TiGraMITe Python package, which code is freely available at https://github.com/jakobrunge/tigramite.

## 4 Results

### 4.1 The Confounding Effect of Stratospheric Autocorrelations

In this section, we study the difference between quantifying the S‐T coupling with lagged correlation or with partial correlation. To facilitate the comparison with the correlation results obtained by Byrne and Shepherd (2018), we structure the statistical analysis in the same way. The anomaly time series are monthly averaged. The statistical coefficients are computed between a measure of PoV (or Jet) at a given month between September and January and a measure of the same variable (or the other) at subsequent months lagged by +1 to *τ*_*max*_=+3; thus, the sample statistics is 38, after excluding the 2002–2003 season. The significance level is *α*=0.05 in a one‐tailed Student *t* distribution for this figure only.

Despite the different proxies used for jet and vortex, the correlation plots in Figure 1a resemble closely those in Byrne and Shepherd (2018) (therein Figures 7, 14a, and 14b). Because the cross correlation with vortex leading jet is larger than the jet autocorrelation, Byrne and Shepherd (2018) use it to argue for the vortex being a better predictor of the jet than the jet itself between October and December, as previously suggested by Gerber et al. (2010). However, such a statement fails to account for the role of the strong vortex autocorrelation, already noticeable in the similarity between the left plots in Figure 1a. Interestingly, the upward correlation is also statistically significant at lag 1 from October to January (not computed in the mentioned articles), which could suggest there is an upward component to the connection too (Figure 1a top‐right).

A conditional analysis results in a different quantification of the coupling (Figure 1b). Between September and December, the vortex adds information to its future state for a maximum of 1‐month lag, while the autocorrelations suggested influence for 2–3 months (compare top‐left plots in Figures 1a and 1b). More strikingly, only a few positive downward links are found compared to the extended cross‐correlation structure: a confident conclusion on the vortex being a tropospheric predictor seems less obvious (compare bottom‐left plots). A similar result holds for the upward connection since the cross‐correlation structure disappears after conditioning.

The reason for the weakening of the coupling lies in the conditioning set of the partial correlation *ρ* ^*MIT*^(*PoV*_*m*_,*Jet*_*m*+*τ*_), and of its upward version. Inspecting all the pairs, we found the set almost solely consisting of the 1‐month vortex precursor: *Z* ^*MIT*^=*PoV*_*m*−1_ (downward) or *PoV*_*m*+*τ*−1_(upward). This is due to the vortex being strongly autocorrelated and thus retained in the *Z* ^*MIT*^ conditions. Given the extent of this weakening, it is evident that the stratospheric autocorrelation accounts for most of the signal manifest in both cross correlations, including the large persistence of the downward correlation. This conclusion can be demonstrated in general terms by deriving the lagged and partial cross correlations of a two‐variable linear system as functions of the auto‐dependence coefficients, as studied in Runge et al. (2014) (see Text S2 in the supporting information for the formulae).

### 4.2 Spring‐to‐Summer Predictors

**Figure 2:**
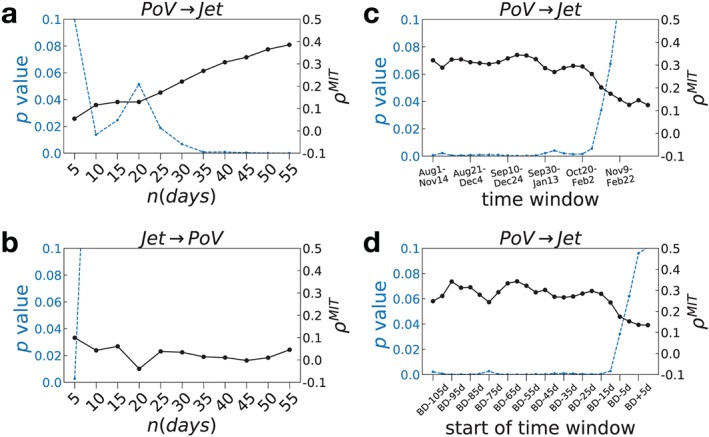
*ρ* ^*MIT*^ (black) and *p* value (blue) for increasing *n*‐averaging and fixed ONDJ time window: (a) downward link and (b) upward link. *ρ* ^*MIT*^ (black) and *p* value (blue) for the downward link at fixed 35‐day aggregation and changing time window of 105 days: (c) fixed calendar dates and (d) relative to BD date. The maximum lag for all the analysis is *τ*_*max*_=3 and the confidence level is kept maximum (*α*=1) to see the changes in *p* value for the various configurations.

The patchy structure of the downward partial correlations suggests that a sample size issue might be at play and potentially hiding the signal. To alleviate this problem, we analyze groups of months rather than individual months, as modeling studies agree that the dynamical coupling persists from roughly September to January (Byrne et al., 2019). First, the timescales at which the upward and downward links come into play are explored. While the multiple months time‐window October through January (ONDJ) is fixed, the averaging block size of the time series is changed: *n*=5,..,55 (with corresponding sample sizes from 912 to 80). The downward link at lag +1 becomes significant and strong for averaging larger than 30 days (*p* value ≤10^−3^, *ρ* ^*MIT*^ ≥ 0.25) (see Figure 2a). We recover the known role of the stratosphere as a source of tropospheric predictability, but the influence needs around one month to be manifest in the troposphere, which suggests a role for lower‐stratospheric radiative processes (Hitchcock et al., 2013 ‐ Figure 1 therein). This timescale is very close to the one used in Section 4.1, so the nonrobust downward link found therein for individual months was most likely a sample size issue. Figure 2b shows that the upward link is detected only below a 10 day average, which seems reasonable given planetary wave propagation timescales. As for same‐variable connections at lag +1, the jet is significantly auto‐dependent only for an average of up to 20 days, while the vortex is so up to 50 days (not shown), consistent with the larger temporal coherence of the stratosphere compared to the troposphere. Links at larger lags appear only for vortex auto‐dependency (+2 only for a tested *τ*_*max*_=+3) but are nonrobust to different averaging (not shown). Figures 2a and 2b suggest that a reasonable intraseasonal timescale to study the S‐T coupling is 35 days. This choice strikes a balance between signal detectability (the need for large enough *n*, Figure 2a ) and data set sample size (small enough *n*). The smooth increase of values in Figure 2a is expected due to the vortex auto‐dependency, which is baked into the downward link as soon as the averaging period becomes larger than the timescale of the direct connection. This consideration adds an independent motivation to choosing the smallest possible *n* at which the coupling is manifest. An alternative time window September through December (SOND) gives similar results.

To study the persistence of the downward link from August to February, we fix the number of days *T* of the time window (105 days) and change the starting day *t*_0_ (from 1 August to 1 December; Figure 2c). An average of 35 days is performed after the time window is selected. The downward link persists from August to January but loses significance when the end of the time window reaches into February: this shows that the vortex decouples from the jet in the summer. To address the effect of the BD date, we perform an alternative time window selection, where *t*_0_ is chosen each year according to the BD date (*t*_0_=*BD*−*n* days; Figure 2d). The BD‐specific downward link is strong and robust when the time window begins well before the BD date and has a rapid drop in significance when *t*_0_ is closer than about 20 days. This provides some evidence that the BD date is an indicator of the end of the coupling period.

According to the above analysis, the best period to evaluate the intraseasonal S‐T coupling is between the beginning of September and mid‐January (ending before February) with a time averaging of 35 days. The sample size achieved is 152 = 4 (samples/year) * 38(years). We evaluate all links up to *τ*_*max*_=+2 at a significance level *α*=0.005, which strongly controls for false positives (see Table S1). The connections detected are two: the downward link, as expected, and the vortex auto‐dependence, and can be written as the linear system:

(1) $$\left\{\begin{array}{ll} {\bar{PoV}}_{t} & =a\;{\bar{PoV}}_{t-1}+\varepsilon_{P,t}\\ {\bar{Jet}}_{t} & =c\;{\bar{PoV}}_{t-1}+\varepsilon_{J,t} \end{array}\right.,$$

where (*ε*_*P*_,*ε*_*J*_)_*t*_ is an independent and identically distributed Gaussian noise of covariance Σ and zero mean. The overline stands for standardized time series and lag 1 corresponds to 35 days. A linear regression of each variable onto the detected standardized predictors gives *a*=0.55±0.11 and *c*=0.37±0.15. The covariance matrix is fitted to the two‐dimensional residuals giving $\Sigma =\left(\begin{array}{ll} 0.70 & 0.35\\ 0.35 & 0.86 \end{array}\right)$. Equation (1) recovers an interaction supported by previous literature as mentioned in the Introduction but offers a more parsimonious description (fewer coefficients than needed to describe the correlations). The vortex explains 37% of the monthly jet variability and can be used as its statistical predictor a month ahead. The jet does not provide a detectable source of information to itself on this timescale, nor to the vortex.

**Figure 3:**
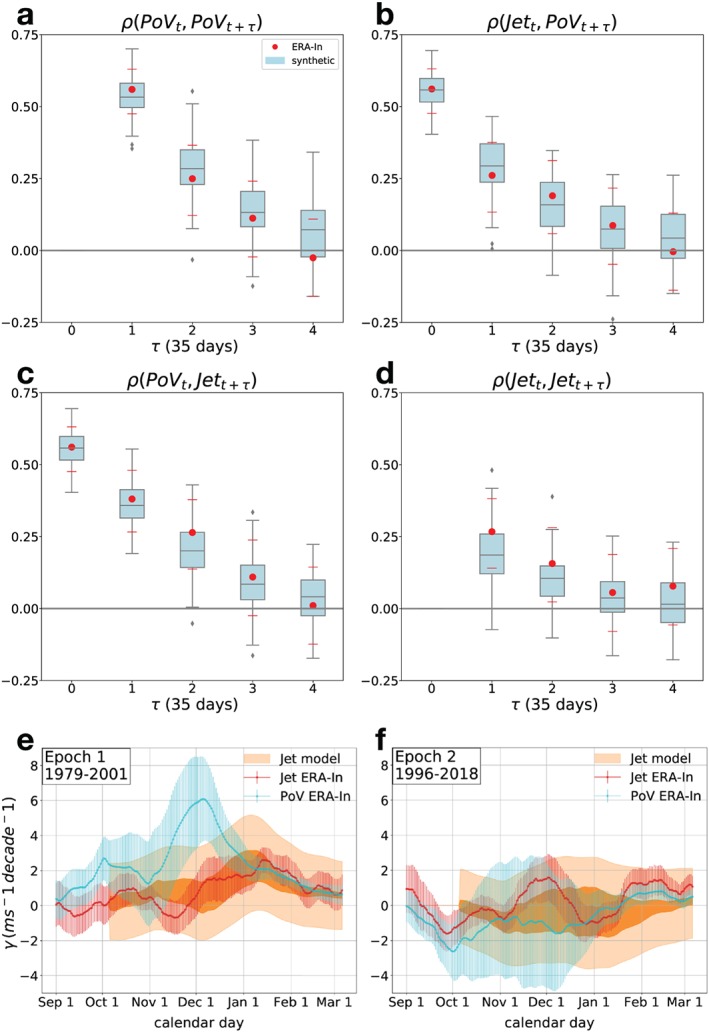
(a)‐(d) Correlations of reanalysis (red dots) and synthetic (blue box plot) time series. 100 synthetic time series of length 152 are generated using Equation (1) and the coefficients derived in Section 4.2. Blue boxes cover the 25–75 percentile ranges; the line is at the median; whiskers show 9–91 percentiles. Red dots are the observational data with bars indicating the 95% confidence range according to a two‐tailed Student *t* distribution. (e) ‐ (f) Trend analysis: PoV and Jet observed trends (blue and red) and their uncertainty computed as described in Section 4.3. The modeled Jet trend and uncertainty (orange) are the ensemble average slope and spread (the latter smoothed using a Gaussian window with a 15‐day sigma width). Because the uncertainty in the observed vortex trend is non‐negligible (large blue error bar, $\sigma_{\gamma_{P}}^{obs}$), we add the associated uncertainty $c\cdot \sigma_{\gamma_{P}}^{obs}$ (dark orange) to the ensemble spread (light orange).

Although the time series are too short to be split into training and testing subsets, we can compare the correlation structure of the model (from data generated using Equation (1)) with the observed one. Since the predictors and coefficients have not been chosen to optimize the resemblance of these features, this comparison provides an independent check on our result. Good agreement is found, meaning that our minimal model can recover the correlation fingerprint of the reanalysis data (Figures 3a‐3d). This suggests that our set of variables is capturing the first‐order relationship between the observed vortex and the jet; that is, there is no major missing explanatory factor on the monthly timescale.

We stress that the jet autocorrelation is also matched despite the absence of direct tropospheric auto‐dependence (no *Jet*_*t*_→*Jet*_*t*+*τ*_ detected; see Table S1). The jet autocorrelation inherits the long timescale from the vortex via the downward link regardless of the fact that the jet itself is not autodependent (see its analytical formula derived from Equation (1) in Text S3). Thus, the enhanced autocorrelation of the tropospheric SAM observed during this period of the year can be attributed to the seasonal stratospheric influence. The effect of a tropospheric eddy feedback could increase the jet autocorrelation timescale further but is not needed to explain the observed values. This may help account for the lack of any relationship between the jet response to climate change and the jet autocorrelation timescale in austral summer (Simpson & Polvani, 2016), which might otherwise be expected if such a timescale reflected mostly the strength of tropospheric feedback processes (Gritsun & Branstator, 2007).

### 4.3 Connection Between Observed Circulation Trends

Equation (1) comes with the implication that an exogenous forcing term added to $\bar{PoV}$, $\tilde{\gamma }t$, would result in $\bar{PoV}$ and $\bar{Jet}$ asymptotically described by the trends $\frac{\tilde{\gamma }}{1-a}t$ and $c\frac{\tilde{\gamma }}{1-a}t$, respectively. The coefficient *c* controls how effectively the externally induced stratospheric trend is transferred to the tropospheric variable.

The strength of the late spring SH polar vortex is indeed known to have increased in the late 20th century, mainly as a result of the cooling of the polar stratosphere due to anthropogenic ozone depletion (McLandress et al., 2010; Waugh et al., 1999). It is also established that the concurrent positive trend in the SAM seen in austral summer is a response to the same cause, with a poleward trend of the tropospheric jet (McLandress et al., 2011) corresponding to a delayed seasonal equatorward shift (Byrne et al., 2017; Sun et al., 2014).

We force Equation (1) with the reanalysis vortex trend, determine the predicted synthetic jet trend, and compare it with the reanalysis jet trend. The reanalysis trends are computed at each calendar day (between 1 September and 5 March) as the increase per decade of the 30‐day mean value of the time series around that calendar day across the years of interest. An ensemble of 100 time series of 30 time steps is generated for each vortex trend value (100 is to estimate the uncertainty and 30 is to be close to the sample used to fit the trend, 22). Since the lag of the downward link is 35 days, the synthetic trend is associated to 35 calendar days later than the forcing. The results discussed in the following also hold if we include a small jet auto‐dependence that could represent a tropospheric feedback that was too weak to be detected by our analysis (adding the term $b{\bar{Jet}}_{t-1}$ to the jet formula of Equation (1) with coefficient *b*=0.1 or 0.2; not shown). We treat the first 22 years of the record, 1979–2001 (Epoch 1), when ozone was declining strongly, separately from the last 22 years, 1996‐2018 (Epoch 2), when the ozone hole was approximately stabilized. The overlapping is chosen to avoid too strong an end‐point interdependence and to create reasonably long epochs of equal length. Details of the calculation are found in Text S4 of the supporting information.

In Epoch 1 (Figure 3e), the observed vortex trend (blue) is strong as expected. The induced synthetic jet trend (orange) overlaps with the observed trend (red). In Epoch 2, no significant stratospheric trend is seen, which induces no synthetic jet trend (Figure 3f). The latter again agrees with the reanalysis, as the observed jet trend is weak and only statistically significant in February. Hence, the linear model seems to have minimal but relevant ingredients that can capture the statistical connection between the vortex and jet long‐term trends. This agrees with the physical idea that a coupling relation based on internal variability can be used to understand the causal links between forced responses (Gritsun & Branstator, 2007). It is also consistent with the relation found by Ceppi and Shepherd (2019) for the SH S‐T coupling for greenhouse gas forcing.

## 5 Conclusions

The direct influence of the SH stratospheric polar vortex on the tropospheric eddy‐driven jet during the spring‐to‐summer transition has been estimated from reanalysis data using a partial correlation analysis. The statistical method, rooted in causal network theory, ensures control of confounding effects, such as the strong stratospheric autocorrelation. The inferred statistical model can provide a minimal description of the coupling, which is physically consistent with previous studies but adds a novel quantitative estimation of its timescale and strength. This quantification can entirely explain the observed jet autocorrelation during this time of year as arising from the influence of the stratosphere. We also give a proof of concept by testing the statistical model against ozone‐induced trends.

The present work adds to the growing body of literature in the field of conditional analysis in climate dynamics (Di Capua et al., 2019; Kretschmer et al., 2017; Samarasinghe et al., 2018). We provide further evidence of its practical advantage in identifying the most parsimonious set of connections in a complex autocorrelated system like S‐T coupling. More generally, the core idea behind the method seems suitable to identify robust connections that can be used for emergent constraints and to perform succinct diagnosis of model error and offers an alternative route to intermodel comparisons (Hammerling et al., 2018; Runge et al., 2019).

## Supporting information



Supporting Information S1
