# Supplementary material for: Quantifying the Timescale and Strength of Southern Hemisphere Intraseasonal Stratosphere‐troposphere Coupling
Source: Geophys Res Lett. 2019 Nov 20;46(22):13479–87. doi: 10.1029/2019GL084763 (PMC6988456; doi:10.1029/2019GL084763)
Supplement: Supplementary file 1 — Supporting Information S1 [file GRL-46-13479-s001.pdf]

Supporting Information for "Quantifying the timescale and strength of Southern Hemisphere intra-seasonal stratosphere-troposphere coupling"

Elena Saggioro[1], Theodore G. Shepherd[2]

[1]Department of Mathematics and Statistics, University of Reading, Reading, UK

[2]Department of Meteorology, University of Reading, Reading, UK

Contents of this file

Text S1 to S4

Table S1

Introduction

Text S1 describes in more detail the causal inference algorithm introduced in the Methods section (Section 3). **Text S2** shows the effect of auto-dependence coefficients on the cross-correlation for a two-variable linear system (Section 4.1). **Text S3** shows the analytical expression for both $\overline{PoV}$ and $\overline{Jet}$ autocorrelation functions derived from Eq. 1

Corresponding author: E. Saggioro, Department of Mathematics and Statistics, University of Reading, JJT Building Room 212, Whiteknights, Reading, RG6 6AX, UK. (e.saggioro@pgr.reading.ac.uk)

(Section 4.2). **Text S4** explains the procedure needed to map the linearly forced time-series, generated using the standardized linear coefficients of Eq. 1, back onto its physical variables (Section 4.3).

Table S1 lists all the links (significant and not) computed in the final analysis of Section 4.2.

Text S1

The algorithm used to produce the present analysis (PCMCI, Runge (2018)) is a modification of the more commonly used causal algorithm (PC, Spirtes and Glymour (1991)) with the additional benefit of providing an estimate of link strength (Runge et al., 2014). The latter property is closely related to the computation of the conditioning set Z^{MIT}.

Given a system of N variables $\mathbf{X}_t$ with $t = 1, \ldots, T$, consider each pair of lagged variables $(X_t, Y_{t+\tau})$ at each lag $\tau = 1, \ldots, \tau_{max}$. To determine which pairs are predictor-predictand (as defined in the main text, Section 3) the partial correlation $\rho(X_t, Y_{t+\tau}|S_{t'<t+\tau})$ has to be computed. By definition the conditioning set is $S_{t'<t+\tau} = \{\mathbf{X}_{t+\tau-1}, \mathbf{X}_{t+\tau-2}, \ldots\} \setminus \{X_t\}$ (up to a maximum lag in the past τ_{max}) and it can be very high dimensional ($N\tau_{max} - 1$). While S is a sufficient set to detect conditional independence, it is usually much larger than necessary. The high dimensionality not only represents a computational cost, but also reduces the detection power and thus the reliability of the partial correlation itself (Runge, 2018).

Since in many cases the true links are sparse, PCMCI operates a preliminary step to extract a low dimensional subset of S. This first step, similar to the PC algorithm, removes irrelevant conditions for each of the N variables by iterative independence testing. In this way, to each variable X is assigned a (generally) low dimensional set of potential predictors $\hat{\mathcal{P}}_X$. The conditioning subset for the pair $(X_t, Y_{t+\tau})$ is therefore the union of both variables' potential predictors: $Z^{MIT}_{X_t,Y_{t+\tau}} = \hat{\mathcal{P}}_{X_t} \cup \hat{\mathcal{P}}_{Y_{t+\tau}} \setminus \{X_t\}$. After this preliminary stage, the algorithm goes through the rejection/estimation step (MCI). The null hypothesis is that each pair is connected. By computing the partial correlation ρ^{MIT}, i.e. with Z^{MIT} as

conditioning set, the null hypothesis of a connection is rejected if the computed p-value is larger than a user defined threshold α. MIT stands for Momentary Information Transfer and refers to the property of the measure being lag-specific (momentary), thus removing autocorrelations. This step allows to reliably quantify the strength of the links.

Compared to PC, this method allows to maximise the detection power of the partial correlation and to give an estimate of the strength of the (significant) links. For a detailed discussion of this result we refer to Runge (2018).

Text S2

The following two-variable linear system, studied in Runge et al. (2014), motivates the conclusion of Section 4.1 in the main text from a general perspective. The system

$$\begin{cases} X_t &= a\, X_{t-1} + \varepsilon_{X,t} \\ Y_t &= c\, X_{t-1} + b\, Y_{t-1} + \varepsilon_{Y,t} \end{cases}$$

describes a stochastic process of two auto-dependent variables (through a, b, with $|a|, |b| < 1$ for stationarity) that are one-way coupled, since X contributes to the state of Y (through c) but not vice-versa. The noise terms are independent and Gaussian with covariance matrix Σ and zero mean. The analytical expression of the lagged cross-correlation $\rho(X_t, Y_{t+\tau}) = f(\tau; a, b, c, \Sigma)$ depends on all coefficients of the system (see Table 2 in Runge et al. (2014)). Relevant to our analysis is that the amplitude and position of its peak – often used as a diagnostic for lagged interaction – can be modulated according to the values of the auto-dependent coefficients a and b. Also, the function f does not markedly decay for a specific sign of the lag, thus potentially suggesting that Y directly influences X. This is because correlation measures the collinearity between lagged time series, which can be due to all sorts of effects. On the other hand, the partial correla-

tion reads $\rho^{MIT}\left(X_t, Y_{t+\tau}\right) = \delta_{\tau,1}\sqrt{\dfrac{c^2\sigma_X^2}{c^2\sigma_X^2+\sigma_Y^2}}$ where $\delta_{\tau,1}$ is the Kronecker delta function, $\sigma_X^2 = \Sigma_{XX}$ and $\sigma_Y^2 = \Sigma_{YY}$ (again Table 2 in Runge et al. (2014)). The effect of both autocorrelations has been removed (no a, b dependence) thanks to the conditioning set $Z^{MIT} = \{X_{t+\tau-1}, Y_{t+\tau-1}, X_{t-1}\}$. The difference between the correlation and partial correlation function is largely due to the auto-dependence coefficients. In the vortex-jet case, the large auto-dependence of the vortex (Fig.1b, top-left) can be seen as analogous to a large coefficient a herein. The fact that conditioning on a $Z^{MIT} = PoV_{m-1}$(or $PoV_{m+\tau-1}$) results in a small downward and upward ρ^{MIT} shows the vortex auto-dependence is indeed responsible for the large and persistent cross-correlations.

Text S3

The autocorrelation of vortex and jet analytically derived from Eq. 1 read:

$$\rho_{\overline{PoV}}(\tau) = a^{|\tau|}$$

$$\rho_{Jet}(\tau) = \frac{1}{c^2\sigma_X^2 + \sigma_Y^2(1-a^2)}\left[c^2\sigma_X^2\rho_{PoV}(\tau) + \delta_{\tau,0}\sigma_Y^2(1-a^2) + (1-\delta_{\tau,0})c(1-a^2)\sigma_{XY}a^{|\tau|-1}\right]$$

where Σ in the main text is here defined entry-wise as $\Sigma = \left(\begin{smallmatrix}\sigma_P^2 & \sigma_{PJ}\\ \sigma_{PJ} & \sigma_J^2\end{smallmatrix}\right)$. The jet autocorrelation function comprises three terms. The first term is proportional to the vortex autocorrelation, thus it inherits the vortex autocorrelation timescale thanks to $c \neq 0$. The second term ensures $\rho_{\overline{Jet}}(0) = 1$. The third term includes the effects of a contemporaneous connection between the two time series ($\sigma_{JP} \neq 0$; physically this can represent processes influencing both vortex and jet on a timescale smaller than $\tau = 1$). The decorrelation time scale of this term is set by $a^{|\tau|-1}$, thus is also dependent on the vortex autocorrelation.

We note that the correlation structure of the time-series generated using Eq. 1 and the

standardised coefficient can be directly compared with the reanalysis correlations because of the property of invariance under linear transformations.

Text S4

The coefficients computed for Eq. 1 in Section 4.2 come from standardised time series. The internal variability of the standardised vortex is $Var[\overline{PoV}] = \sigma_P^2/(1-a^2)$, according to Eq. 1 (to estimate it we use coefficients calculated in Section 4.2). To ensure that the trending synthetic vortex time-series is appropriately scaled we need to impose that it has the same variability-to-trend ratio as the observed vortex. The ratio is $R =$ vortex variability/ strength increase due to the trend. Keeping $Var[\overline{PoV}]$ fixed while imposing $R^{obs} = R^{synth}$, this results in the synthetic trend coefficient being $\tilde{\gamma} = K \cdot {\gamma_P}^{obs}$ with the factor

$$K = \left[(1-a) \frac{T^{obs}}{T^{synth}} \frac{\sqrt{\sigma_P^2/(1-a^2)}}{\sqrt{Var[\text{PoV}]}} \right].$$

Note that K depends only on vortex parameters. In particular, the observed jet trend is not used in any way. Specifically, $R^{obs} = \sqrt{Var[\text{PoV}]}/T^{obs}{\gamma_P}^{obs}$, with ${\gamma_P}^{obs}(ms^{-1}/month)$ obtained from reanalysis as described in Section 4.3, T^{obs} =22(years in one Epoch)$\cdot$ 12 (months in a year) and $Var[\text{PoV}]$ in the variance of the PoV monthly anomalies from reanalysis. $R^{synth} = \sqrt{Var[\overline{PoV}]}/T^{synth}\tilde{\gamma}$, with $T^{synth} = 30$ time steps (an arbitrary length chosen to be close to the small sample size from which each calendar day-specific reanalysis trend is estimated). Because the analytic prediction for the synthetic jet trend is $\tilde{\gamma}c/(1-a)$, we can convert it to physical values by K^{-1} multiplication.

References

Runge, J., Petoukhov, V., & Kurths, J. (2014). Quantifying the strength and delay of climatic

interactions: The ambiguities of cross correlation and a novel measure based on graphical models. *Journal of Climate*, *27*(2), 720-739. doi: 10.1175/jcli-d-13-00159.1

Runge, J. (2018). Causal network reconstruction from time series: From theoretical assumptions to practical estimation. *Chaos: An Interdisciplinary Journal of Nonlinear Science*, *28*(7), 075310. doi: 10.1063/1.5025050

Spirtes, P., & Glymour, C. (1991). An algorithm for fast recovery of sparse causal graphs. *Social Science Computer Review*, *9*(1), 62–72. doi: 10.1177/089443939100900106

Table S1. PCMCI results for n = 35 days, time window = Sept 1 to Jan 19 ; PCMCI parameters: $\alpha = 0.005$, $\tau_{max} = +2$. The statistically significant links are indicated in bold font.

Link	lag	ρ^{MIT}	95% conf.	p-value
**PoV → PoV**	**+1**	**0.56**	**(0.472, 0.628)**	**1.5**$\cdot 10^{-5}$
**PoV → Jet**	**+1**	**0.29**	**(0.161, 0.399)**	**1.0**$\cdot 10^{-3}$
Jet → Jet	+1	0.063	(-0.072, 0.196)	0.44
Jet → PoV	+1	-0.064	(-0.197, 0.071)	0.43
PoV → PoV	+2	-0.14	(-0.272, -0.009)	0.07
PoV → Jet	+2	0.06	(-0.069, 0.200)	0.41
Jet → Jet	+2	0.06	(-0.071, 0.197)	0.43
Jet → PoV	+2	0.14	(0.008, 0.271)	0.08
